# Comparison of Antiviral Activity of Gemcitabine with 2′-Fluoro-2′-Deoxycytidine and Combination Therapy with Remdesivir against SARS-CoV-2

**DOI:** 10.3390/ijms22041581

**Published:** 2021-02-04

**Authors:** Yejin Jang, Jin Soo Shin, Myoung Kyu Lee, Eunhye Jung, Timothy An, Uk-Il Kim, Kyungjin Kim, Meehyein Kim

**Affiliations:** 1 Infectious Diseases Therapeutic Research Center, Korea Research Institute of Chemical Technology (KRICT), Daejeon 34114, Korea; erythro1@krict.re.kr (Y.J.); jsshin@krict.re.kr (J.S.S.); lmg1671@krict.re.kr (M.K.L.); ehjung@krict.re.kr (E.J.); timothy@krict.re.kr (T.A.); 2 Graduate School of New Drug Discovery and Development, Chungnam National University, Daejeon 34134, Korea; 3 Research and Development Center, ST Pharm Co., Ltd., Seoul 01694, Korea; ukil911@stpharm.co.kr

**Keywords:** SARS-CoV-2, antiviral activity, gemcitabine, 2′-fluoro-2′-deoxycytidine

## Abstract

Severe acute respiratory syndrome coronavirus 2 (SARS-CoV-2) is the causative agent of the coronavirus disease 2019 (COVID-19) pandemic. The virus still spreads globally through human-to-human transmission. Nevertheless, there are no specific treatments clinically approved. This study aimed to compare antiviral activity of gemcitabine and its analogue 2′-fluoro-2′-deoxycytidine (2FdC) against SARS-CoV-2 as well as cytotoxicity in vitro. Fluorescent image-based antiviral assays revealed that gemcitabine was highly potent, with a 50% effective concentration (EC_50_) of 1.2 μM, more active than the well-known nucleoside monophosphate remdesivir (EC_50_ = 35.4 μM). In contrast, 2FdC was marginally active (EC_50_ = 175.2 μM). For all three compounds, the 50% cytotoxic concentration (CC_50_) values were over 300 μM toward Vero CCL-81 cells. Western blot and quantitative reverse-transcription polymerase chain reaction analyses verified that gemcitabine blocked viral protein expression in virus-infected cells, not only Vero CCL-81 cells but also Calu-3 human lung epithelial cells in a dose-dependent manner. It was found that gemcitabine has a synergistic effect when combined with remdesivir. This report suggests that the difluoro group of gemcitabine is critical for the antiviral activity and that its combination with other evaluated antiviral drugs, such as remdesivir, could be a desirable option to treat SARS-CoV-2 infection.

## 1. Introduction

Coronaviruses (CoVs) belong to the family *Coronaviridae* and are organized into four groups: α-, β-, γ-, and δ-CoVs [1]. These enveloped viruses have a positive-sense genome of single-stranded RNA ranging from 26 to 32 kilobase (kb), encoding 16 non-structural and four structural proteins comprising spike (S), nucleocapsid (N), membrane (M), and envelope (E) proteins. CoVs had been known to be responsible for milder infections such as the common cold. However, recently lethal viral variants have emerged, that caused severe acute respiratory syndrome (SARS), Middle East respiratory syndrome (MERS), and coronavirus disease 2019 (COVID-19) [2,3].

SARS coronavirus 2 (SARS-CoV-2) originated in Wuhan, China, in December 2019, and spread rapidly worldwide [4,5]. Since official declaration of COVID-19 outbreak as a pandemic by World Health Organization (WHO) on January 2020, new infections or deaths are still recorded daily. Despite the rapid transmission rate and devastating mortality, CoV-specific antivirals are not yet available. Several research groups have attempted drug repurposing therapeutic strategies using chemical libraries composed of US Food and Drug Administration (FDA)-approved drugs to treat SARS-CoV-2 infection. They suggested that remdesivir (also named GS-5734), (hydroxy)chloroquine, ciclesonide, and some other compounds known to target other diseases may be effective against the virus, providing experimental evidence for initiating clinical studies and for authorizing emergency use of remdesivir [6,7].

On the basis of viral life cycle, this virus enters the target cells mediated by two main cellular proteins, angiotensin-converting enzyme 2 (ACE2) and transmembrane serine protease 2 (TMPRSS2) that senses the receptor-binding domain (RBD) of S and activates the viral glycoprotein [8,9], respectively. Endosome-virus membrane fusion or direct fusion between plasma-virus membranes facilitates release of viral RNA into the cytoplasm [10,11], of which double-membrane vesicles (DMVs) provide a place for viral RNA replication and transcription from the genomic RNA through enzymatic function of RNA-dependent RNA polymerase (RdRp) and helicase (named nsp13). Activity of viral RdRp is attributed to the catalytic component, non-structural protein (nsp12), and two accessary components, nsp7 and nsp8 [12,13]. More recently, cryo-electron microscopic analyses elucidated that the triphosphated form of remdesivir is bound to the incoming nucleoside triphosphate (NTP) substrate recognition site within nsp12 [14,15].

**Figure 1:**
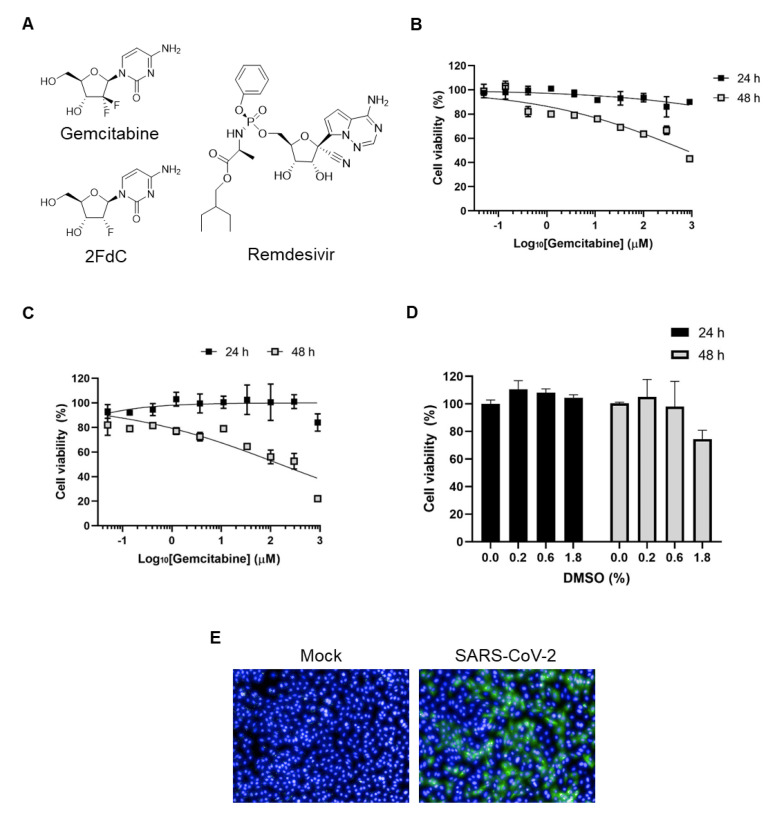
Chemical structures of the compounds tested and visualization of SARS-CoV-2-infected Vero cells. (**A**) Chemical structures of gemcitabine, 2FdC, and remdesivir. (**B**) MTT-based cytotoxicity assay of gemcitabine at different concentrations and time points. Vero cells were treated with increasing concentrations of gemcitabine for 24 h (black square) or 48 h (gray square). Percentage cell viability was measured by using MTT, in which mock-treated cells served as a control (100%). (**C**) Fluorescein diacetate-based cytotoxicity assay of gemcitabine. Vero cells were treated with increasing concentrations of gemcitabine for 24 (black square) and 48 h (gray square). Percentage cell viability was measured by addition of fluorescein diacetate, in which mock-treated cells served as a control (100%). (**D**) Fluorescein diacetate-based cytotoxicity assay of a delivery vehicle. Increasing concentrations of DMSO, 0.2, 0.6 and 1.8% (*v*/*v*), that were identically included in 100, 300 and 900 μM gemcitabine shown in (**C**), were treated to Vero cells for 24 (black bar) and 48 h (gray bar). Values in (**B**–**D**) are means ± standard deviations from thee three independent experiments. (**E**) Visualization of SARS-CoV-2 infection. Vero cells were mock-infected (Mock) or infected with SARS-CoV-2 at a multiplicity of infection (MOI) of 0.02 for 24 h. Viral spike (S) protein was probed with mouse anti-S antibody and Alexa Fluor 488-conjugated goat anti-mouse antibody (green). Cellular nuclei were counterstained with DAPI (blue). Magnification ×20.

Deoxycytidine nucleoside analogues, 2′,2′-difluoro-2′-dexocycytidine (gemcitabine) and 2′-fluoro-2′-deoxycytidine (2FdC), have been identified as broad-spectrum antiviral compounds (Figure 1A). Particularly, gemcitabine has been reported to block infection of diverse DNA and RNA viruses, including herpes simplex virus type 1, Zika virus, influenza virus, CoV, and enteroviruses [16,17,18,19]. Its activity against SARS-CoV-2 has been recently reported in Vero E6 and Huh7 cells, but not yet extensively investigated particularly in terms of structure-and-activity relationship or combination with other antivirals [20]. Meanwhile, 2FdC was elucidated to be active against influenza virus and bunyaviruses in cells and a mouse model, respectively [21,22]. Like SARS-CoV and MERS-CoV, SARS-CoV-2 belongs the β-CoV genus but has different pathological features such as transmissibility, mortality, genome structure and even immune responses [23,24]. It addresses why re-evaluation of existing anti-CoV compounds including gemcitabine and 2FdC against SARS-CoV-2 is needed. In the present study, we compared the antiviral and cytotoxic effects of gemcitabine with those of 2FdC in SARS-CoV-2-infected cells by using remdesivir, a monophosphate prodrug of an adenosine nucleoside analogue, as a control. We also examined synergistic effects of gemcitabine or 2FdC with remdesivir. This study could be helpful for designing and developing gemcitabine or its chemical analogues for the treatment of COVID-19 infection.

## 2. Results

### 2.1. Comparison of Antiviral Activity between Gemcitabine and 2FdC Against SARS-CoV-2

Gemcitabine is a widely used anti-cancer drug that has been known to function via incorporation into DNA mainly or RNA. In detail, it affects DNA synthesis by inducing chain termination or starving competing deoxyribonucleotide pools by inhibition of ribonucleotide reductase. In another way, gemcitabine together with its metabolized deaminated compound, 2′,2′-difluoro-2′-dexocyuridine (dFdU) also disturb RNA transcription [25]. These mechanisms are closely related to toxicity of gemcitabine in several cell types [26,27,28]. Accordingly, prior to initiation of antiviral research, it was needed to determine a concentration range or an incubation period, in which cells are able to tolerate exposure to the compound. The result from 3-(4,5-dimethylthiazol-2-yl)-2,5-diphenyl tetrazolium bromide (MTT) assay showed that a 24-h incubation led to minimal cytotoxicity, remaining over 90% of Vero CCL-81 (Vero) cells viable at a maximum concentration of 900 μM (Figure 1B). However, a 48-h incubation caused a dose-dependent decrease in cell viability, resulting in a CC_50_ value of 758.4 ± 1.6 μM. This incubation time- and concentration-dependent toxicity profile of gemcitabine was reproduced in an irrelevant method where fluorescein diacetate was used as a substrate [29] (Figure 1C). The data on day 2 revealed more severe cytotoxicity with only 20% viable at the maximum concentration, 900 μM, of gemcitabine, resulting in a CC_50_ value of 158.0 ± 1.6 μM. Examination of delivery vehicle-derived toxicity showed that this cytotoxicity was induced partially from 1.8% DMSO at the maximum compound concentration but wholly from gemcitabine at lower concentrations (Figure 1D). Actually, assessing antiviral function under subtoxic concentrations is highly important for excluding misinterpretation of the toxicity-derived inhibitory effect. In parallel, we investigated whether the one-day incubation is available for visualization of infected cells in an immunofluorescence assay. To test this, Vero cells were infected with SARS-CoV-2 at an MOI of 0.02 for 24 h. Given well-distributed viral infection in wide-field fluorescent images, the immunofluorescence microscopy confirmed that the one-day incubation is sufficient for quantifying viral infection (Figure 1E). On the basis of both acceptable cell viability and considerable infectivity, all antiviral experiments with gemcitabine were done by its treatment within 24 h hereinafter.

### 2.2. Reduction of Viral Protein and RNA Levels by Gemcitabine in Vero Cells

**Figure 2:**
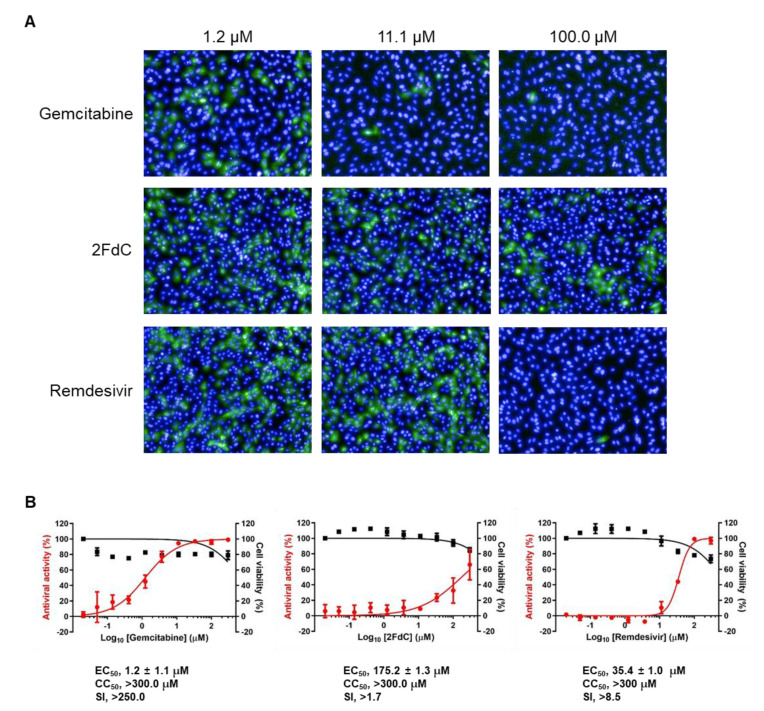
Evaluation of the antiviral activity of gemcitabine, 2FdC, and remdesivir against SARS-CoV-2 in a fluorescence image-based antiviral assay system. (**A**) Visualization of decrease in viral S protein level in the presence of the antiviral compounds. Vero cells were treated with 3-fold serial dilutions of each compound (300 to 0.02 μM over 10 concentrations) for 30 min. Cells were infected with SARS-CoV-2 at an MOI of 0.02. At 24 h post-infection, viral S protein (green) and nuclei (blue) were visualized by fluorescence microscopy. Representative images are shown from eight images per sample at compound concentrations of 1.2, 11.1, and 100.0 μM. Magnification ×20. (**B**) Antiviral activity (left y-axis, red circles) and cell viability (right y-axis, black squares) at increasing concentrations of gemcitabine (left panel), 2FdC (middle panel), and remdesivir (right panel). Percentage means and standard deviations are calculated from four different spots per sample in triplicate. EC_50_ and CC_50_ values were determined from a nonlinear regression equation and are shown below each panel. Selectivity index (SI), ratio of CC_50_ to EC_50_.

To test the antiviral activity of gemcitabine and 2FdC against SARS-CoV-2, the image-based antiviral assay was carried out by addition of 3-fold serial dilutions (from 300 to 0.02 μM at 10 concentration points) of each compound to Vero cells for 30 min before infection (MOI, 0.02), in which remdesivir was used as a control. The resulting microscopic images from the viral S protein and nuclear condensation represented antiviral efficacy and cytotoxicity, respectively (Figure 2A). Gemcitabine potently suppressed viral infection in a dose-dependent manner with little cytotoxicity at the maximal concentration (300 μM), equating to EC_50_ of 1.2 ± 1.1 μM, CC_50_ > 300 μM, and an SI value > 250.0 (Figure 2B). In contrast, 2FdC exhibited weaker antiviral activity with EC_50_ of 175.2 ± 1.3 μM, CC_50_ > 300.0 μM, and SI > 1.7. As expected, remdesivir induced a considerable antiviral effect (EC_50_ of 35.4 ± 1.0 μM, CC_50_ > 300.0 μM, and SI > 8.5), confirming the reliability of the antiviral assay system. The results suggested that gemcitabine is a highly potent antiviral compound inhibiting SARS-CoV-2 replication in vitro, while deletion of one fluorine from gemcitabine causes drastic reduction of antiviral efficacy, stressing that difluoro substitution on position 2′ of the deoxycytidine nucleoside analogue confers antiviral efficacy.

**Figure 3:**
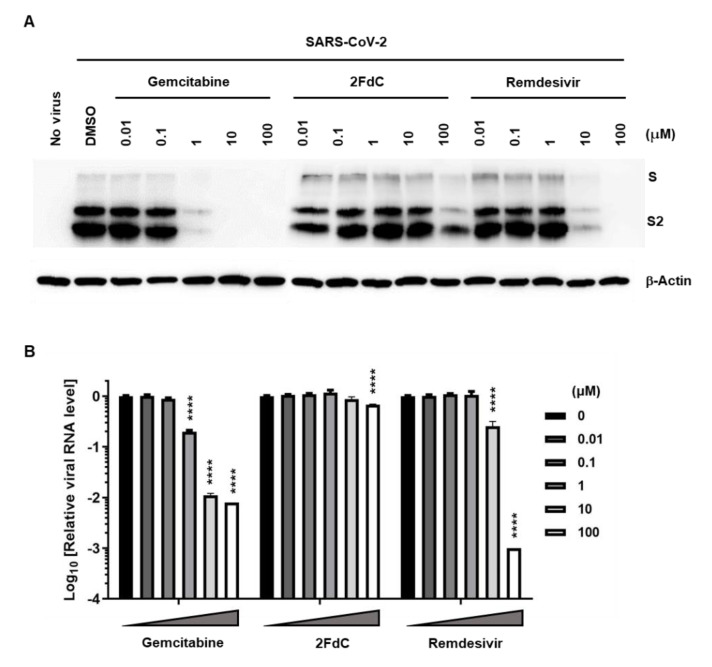
Reduction of viral S protein and viral RNA levels in Vero cells by gemcitabine. (**A**) Western blot analysis probing the S protein. Vero cells in 6-well plates were mock-infected (No virus) or infected with SARS-CoV-2 at an MOI of 0.001 for 1 h at 37 °C. Cells were treated with 10-fold increasing concentrations of gemcitabine, 2FdC, or remdesivir or with a delivery vehicle (0.2% DMSO) for 24 h. Cell lysates were harvested and subjected to 10% SDS-PAGE by loading 30 μg total protein per well. Full-length S and its cleaved product S2 were visualized using an S-specific antibody (upper panel), with β-actin serving as a loading control (lower panel). Both proteins are labeled on the right side of the gels. (**B**) Real-time RT-PCR was performed using culture supernatants from the same samples used for western blot analysis in (**A**). Viral RNA was amplified using an one-step RT-PCR kit targeting the N gene. The RNA titer from virus-infected, 0.2% DMSO-treated sample was used as a mock control, being set as 1. Changes in viral RNA level are depicted using a log scale from cycle threshold (Ct) values. Values are means ± standard deviations from thee three independent experiments. Multiple comparisons were performed between each test group and the control group by two-way analysis of variance (ANOVA). **** *p* < 0.0001.

Even though this image-based screening approach is a relatively robust method for quantitative estimation of antiviral efficacy, it was difficult to exclude the possibility that fluorescent signals could be derived from non-specific binding between antibodies and unidentified nontarget proteins or that their reduction could be affected by an undesirable photon quenching effect from a treated compound. Thus, it was needed to clarify at the molecular level that the decreased immunofluorescence signal in the presence of the compounds was indeed representing reduction of the viral S protein. In addition, we wondered whether the intracellular antiviral function could lead to inhibition of progeny virus secretion into the culture supernatant. To address these critical questions, we conducted western blot analysis using cell lysates and quantitative reverse-transcription PCR (RT-PCR) using culture supernatants. Consistent with the fluorescence microscopic images (Figure 2), immunoblot clearly showed that the viral protein was more markedly reduced by gemcitabine than 2FdC or even remdesivir in Vero cells (Figure 3A). Notably, viral S protein was hardly detected in the presence of 1, 10 and 100 μM gemcitabine, whereas 2FdC barely achieved a considerable reduction at 100 μM and remdesivir exhibited apparent inhibition at 10 and 100 μM. Viral RNA was purified from the culture supernatant at the same time point, i.e., 24 h post-infection. The real-time RT-PCR data revealed that the amount of viral RNA was decreased to 19.8%, 1.1%, and 0.8% in the presence of 1, 10, and 100 μM gemcitabine, respectively (Figure 3B). Similarly, remdesivir suppressed the N gene level to 25.3% and 0.1% at 10 and 100 μM, while only the maximum concentration of 2FdC (100 μM) yielded a marginal reduction in viral RNA level to 68.2%. Taken together, these results proved efficient reductions in both viral protein and RNA levels by gemcitabine, suggesting that this compound has the ability not only to inhibit SARS-CoV-2 replication in cells, but also to block the release of viral progeny to the cell culture supernatants.

### 2.3. Antiviral Activity of Gemcitabine in an Incubation Time-Dependent Manner

**Figure 4:**
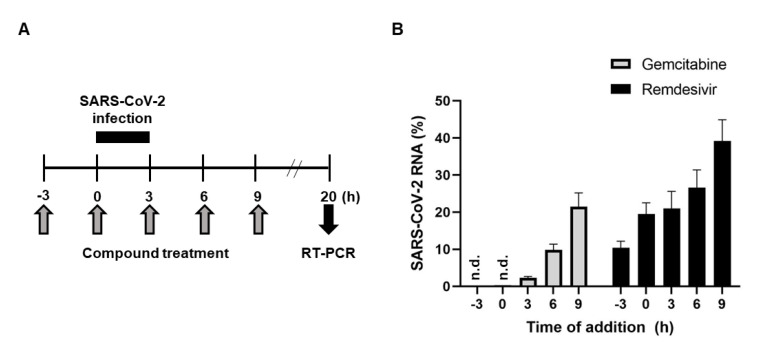
Time-of-addition experiment of gemcitabine. (**A**) Schematic presentation of the time course of viral infection and the compound treatment. (**B**) Quantitative RT-PCR for detecting the SARS-CoV-2 N gene. Vero cells seeded in 24 wells were infected with SARS-CoV-2 at an MOI of 0.01 for 3 h, followed by washing with PBS. In parallel, 10 μM of gemcitabine (gray bars) or remdesivir (black bars) as a control was added at 3-h intervals starting from 3 h before viral infection. At 20 h post-infection, their culture supernatants were harvested for viral RNA preparation and real-time RT-PCR. RNA quantities were calculated relative to the amount from virus-infected, mock compound-treated cells, which was arbitrarily set as 100%. Data represent means and standard deviations from three independent experiments. n.d., not detected.

Time-of-addition experiment was performed to determine which step of the virus life cycle is targeted by gemcitabine. Ten micromolar concentration of the compound was treated independently at 3-h intervals starting from 3 h before viral infection by using another nucleoside analogue, remdesivir, as a control (Figure 4). To remove background noises from the input virus on viral RNA quantitation, SARS-CoV-2 was infected for 3 h conditionally and washed out using phosphate-buffered saline (PBS). Quantitative analysis of viral RNA from the supernatants exhibited that incubation period of gemcitabine was well correlated with its inhibitory efficiency in a similar way as shown with remdesivir. It could be deduced that antiviral activity of gemcitabine appears in an incubation time-dependent manner, potentially being involved in the viral RNA replication step as remdesivir.

**Figure 5:**
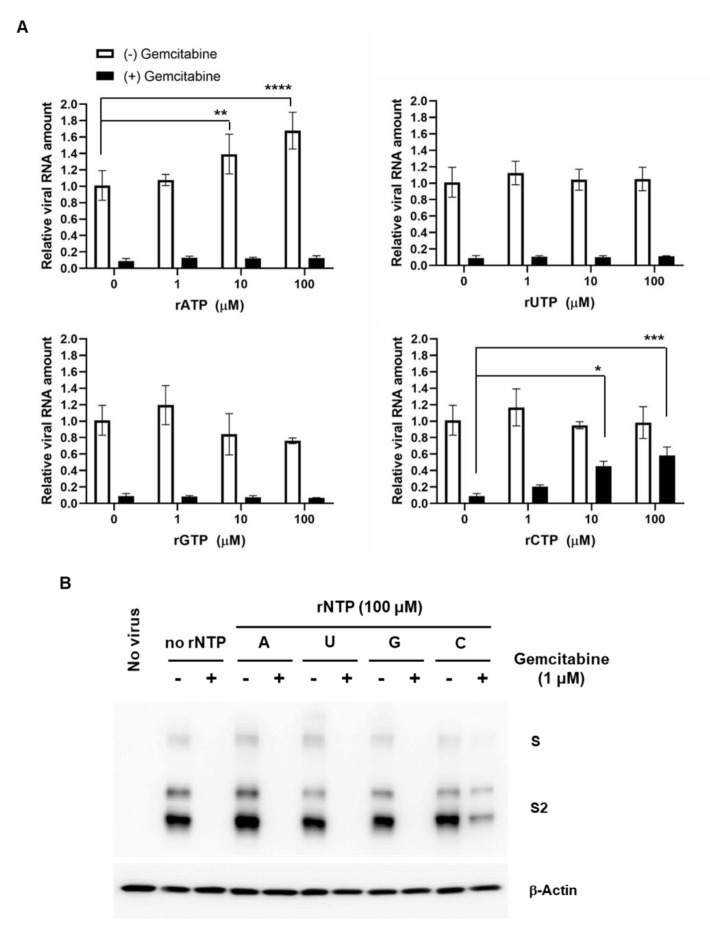
Competition assay between gemcitabine and each rNTP. (**A**) Real-time RT-PCR for measuring viral RNA amounts. Vero cells seeded in 6-well plates were infected with SARS-CoV-2 at an MOI of 0.001 and then treated with increasing concentrations of one of rNTPs including rATP, rUTP, rGTP and rCTP (1, 10, and 100 μM) in the absence (white bars) or presence of gemcitabine (1 μM; black bars) for 24 h. Culture supernatants were harvested for RT-PCR with primers specific for the viral N gene. Infected cells mock-treated or treated with gemcitabine only were used as positive (100%) and negative controls, respectively. Data represent means and standard deviations from three independent experiments. Multiple comparisons were performed between each test group and the rNTP-untreated controls by two-way ANOVA. * *p* < 0.05; ** *p* < 0.01; *** *p* < 0.001; **** *p* < 0.0001. (**B**) Western blot analysis for visualization of viral S protein. The cell lysates prepared for (**A**) with 100 μM rNPTs in the absence (−) or presence of 1 μM gemcitabine (+) were harvested for immunoblotting with anti-S antibody by using β-actin as a loading control. ‘No virus’ means a naive sample without SARS-CoV-2 infection.

It was explored which rNTP is abnormally substituted with gemcitabine during viral RNA synthesis. Although a previous report mentioned that gemcitabine inhibits SARS-CoV-2 infection in a competitive way with rCTP, dose-dependency as well as the roles of other purine nucleosides, rATP and rGTP, were not fully elucidated [20]. Here, antiviral assay was repeated by addition of increasing concentrations of each of four rNTPs (1, 10 and 100 μM) in the absence or presence of gemcitabine (1 μM). Real-time RT-PCR on day 1 revealed that rATP rather stimulated viral replication but no effect when gemcitabine elicits its inhibitory function (Figure 5A, upper left). The finding was not weird when it is considered that affinity of helicase, a key element for viral RNA replication, to the duplex RNA substrate is enhanced at high rATP concentrations [30]. rUTP and rGTP showed no influence on viral replication or on inhibitory potency of gemcitabine (Figure 5A, upper right and lower left, respectively). In consistent with the previous report, gemcitabine-mediated suppression of SARS-CoV-2 infection was relieved by rCTP in a dose-dependent manner (Figure 5A, lower right). This finding was evaluated again by western blot analysis using cell lysates (Figure 5B). Interestingly, 100-fold higher concentration of rCTP was not enough to completely nullify gemcitabine-mediated inhibitory effect, reflecting potential existence of alternative antiviral mechanism that may be associated with host DNA synthesis. Our result suggested that gemcitabine works as an anti-SARS-CoV-2 agent through competition with rCTP, ensuring its participation in the RNA synthesis step.

### 2.4. Inhibition of SARS-CoV-2 Replication in Calu-3 Human Lung Epithelial Cells

**Figure 6:**
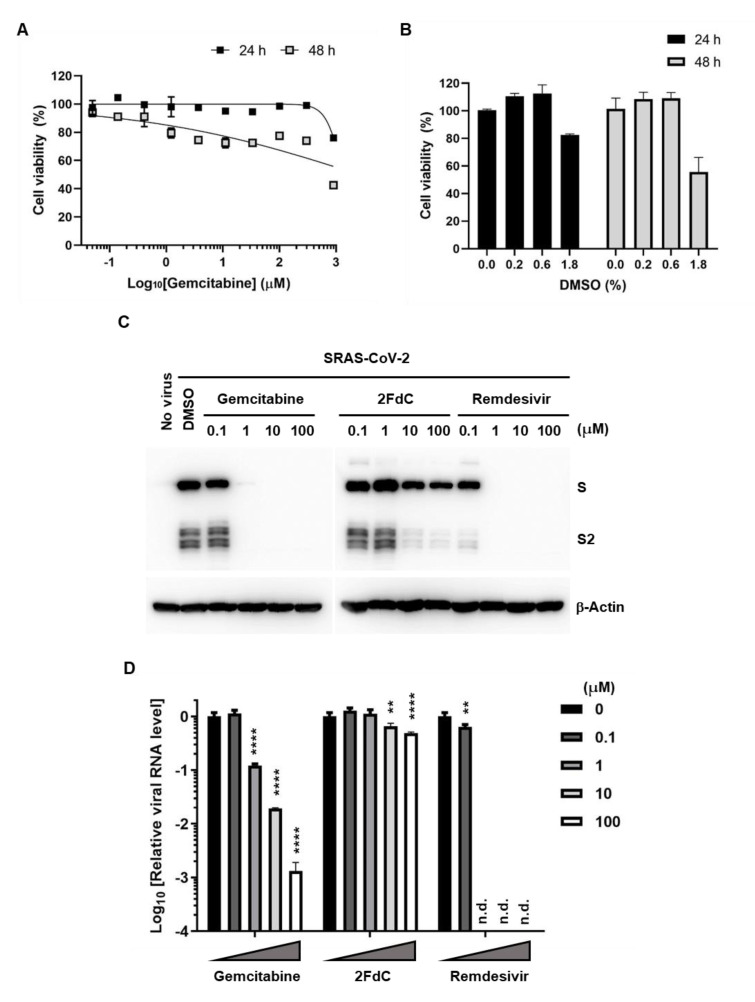
Reduction of viral S protein and viral RNA levels in Calu-3 cells by gemcitabine. (**A**) Cytotoxicity of gemcitabine to Calu-3 cells. Cells were treated with increasing concentrations of gemcitabine for 24 (black square) and 48 h (gray square). Percentage cell viability was measured by MTT assay, in which mock-treated cells served as a control (100%). (**B**) Effect of DMSO on cell viability. Increasing concentrations of DMSO, 0.2, 0.6 and 1.8% (*v*/*v*), that were identically included in 100, 300 and 900 μM gemcitabine shown in (**A**), were treated to Calu-3 cells for 24 (black bars) and 48 h (gray bars) for cell viability assay. Values are means ± standard deviations from thee three independent experiments in (**A**,**B**). (**C**) Western blot of the S protein. Calu-3 cells in 6-well plates were mock-infected (No virus) or infected with SARS-CoV-2 at an MOI of 0.002 for 1 h at 37 °C. Cells were treated with 10-fold increasing concentrations of gemcitabine, 2FdC, or remdesivir or with 0.2% DMSO. On the next day, cell lysates were subjected to 10% SDS-PAGE and immunoblotting. Full-length S and its cleaved product S2 were visualized using an S-specific antibody (upper panel), with β-actin serving as a loading control (lower panel). (**D**) Real-time RT-PCR was performed using culture supernatants from the same samples used in (**A**) by targeting the viral N gene. The RNA titer from virus-infected samples but not treated with any antiviral compound (DMSO) was used as a mock control. Changes in viral RNA level are depicted using a log scale. Values are means ± standard deviations from thee three independent experiments. Multiple comparisons were performed between each test group and the mock control group by two-way analysis of variance (ANOVA). ** *p* < 0.01; **** *p* < 0.0001. n.d., not detected.

Given that main infection sites of SARS-CoV-2 in humans are distributed along the respiratory tract by occupying ciliated airway cells and alveolar type 2 (AT-2) cells as primary targets [31], it was needed to verify whether the antiviral activity of gemcitabine is reproducible in a lung cell line, Calu-3. Before antiviral assay, we examined again cytotoxicity of gemcitabine to the lung epithelial cells on days 1 and 2. Similar to that observed in Vero cells (Figure 1B,C), 24-h incubation did not induce gemcitabine-mediated cytotoxicity, while 48-h incubation resulted in 30~60% cell death at its concentrations above 1.2 μM (Figure 6A). DMSO-derived cytotoxicity was observed only at the highest concentration of gemcitabine (900 μM) that includes 1.8% (*v*/*v*) DMSO (Figure 6B). They collectively indicated availability of antiviral assay on day 1.

Calu-3 cells infected with SARS-CoV-2 at an MOI of 0.002 were treated increasing concentrations of three compounds individually, including gemcitabine, 2FdC and remdesivir. Both western blot analysis and RT-PCR consistently displayed significant antiviral efficacy of gemcitabine, requiring a minimum concentration of 1 μM (Figure 6C,D). Taken together, it can be addressed that antiviral activity of gemcitabine is reproducible in human lung epithelial cells. In contrast to that, 2FdC showed only marginal inhibitory effect. Moreover, it was interesting that viral protein expression in Calu-3 cell lysates as well as viral RNA load in the culture supernatants were more drastically reduced by remdesivir, when compared to the results from Vero cells (compare Figure 3 and Figure 6).

### 2.5. Synergistic Antiviral Effect of Gemcitabine and Remdesivir

**Figure 7:**
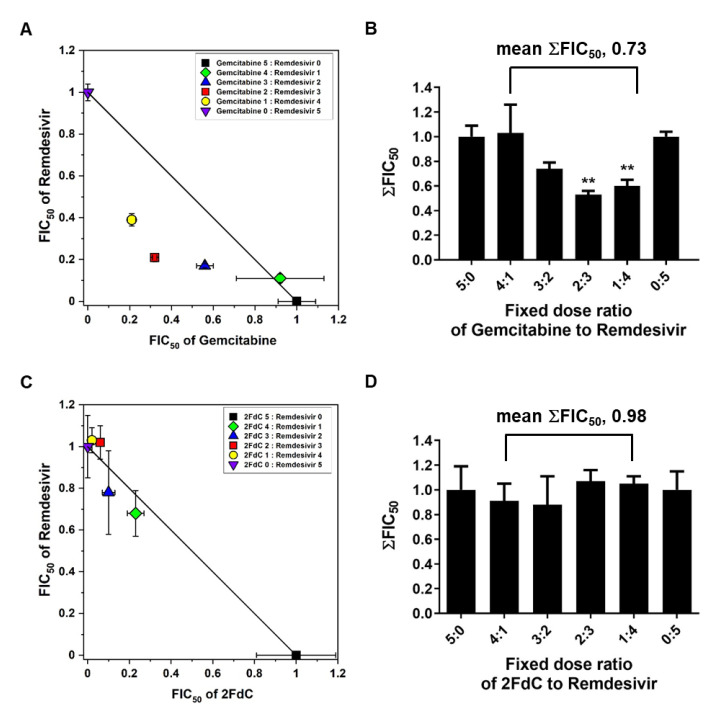
Synergistic effect of gemcitabine with remdesivir. (**A**) Isobologram showing the synergistic interaction between gemcitabine and remdesivir for inhibition of SARS-CoV-2 infection in Vero cells. It was plotted using the relative FIC_50_ values of gemcitabine on the horizontal axis and the values of remdesivir on the vertical axis. (**B**) The sums of both FIC_50_ values (ΣFIC_50_s) at the combination ratios of gemcitabine to remdesivir, 4:1, 3:2, 2:3 and 1:4, and their mean values were quantified. (**C**) Isobologram of the interaction between 2FdC and remdesivir against SARS-CoV-2 in Vero cells. (**D**) The sums of both FIC_50_ values (ΣFIC_50_s) at the fixed combination ratios of 2FdC to remdesivir, 4:1, 3:2, 2:3 and 1:4, and their mean value were quantified. Values are means ± standard deviations from thee three independent experiments. ** *p* < 0.01.

Being different from remdesivir which is an RNA-based adenosine monophosphate analogue, gemcitabine is a DNA-based cytidine analogue. Recognition of these chemical structural features motivated us to investigate whether combination of the two compounds could produce a synergistic effect or an antagonistic effect on inhibition of SARS-CoV-2. Isobologram analysis was performed by their co-treatment at various ratios to virus-infected Vero cells. The result revealed that combination of gemcitabine and remdesivir interacted synergistically, on the basis of the mean of ΣFIC_50_s below 0.8, i.e., 0.73 (Figure 7A,B). Synergistic antiviral effect was maximized at the ratios of gemcitabine to remdesivir to be 2:3 or 1:4. However, combination of 2FdC and remdesivir failed to induce synergistic effect. Their interaction was classified as additive because the mean value of ΣFIC_50_s was close to 1.00, being 0.98 (Figure 7C,D). In respect of antiviral efficacy, the data supported that co-treatment of gemcitabine or its derivatives with remdesivir could be a desirable option for further therapeutic applications.

## 3. Discussion

Here, we investigated the antiviral activity of gemcitabine and 2FdC in a fluorescent image-based cell culture assay system using SARS-CoV-2 isolated from a patient. Treatment of infected cells, either Vero or Calu-3 cells, reproducibly proved that gemcitabine has ability to suppress viral infection more potently than 2FdC which resulted in marginal efficacy (Figure 2, Figure 3 and Figure 6). It is intriguing that chemical structures of 2FdC and gemcitabine are discriminated only by the number of fluorine at the 3′ position on the five-membered sugar ring, where 2FdC has one while gemcitabine has two (Figure 1A). Stark differences in their antiviral activity informed that the difluoro group of the cytidine analogue is one of the critical elements for determining its biological characteristics. Currently, we are devoting to chemical modifications of gemcitabine by focusing on the difluoro substituents for a systematic investigation on structure-and-activity relationship, eventually not only to improve its antiviral activity against SARS-CoV-2 but also to ameliorate cytotoxicity detected at 48 h after treatment (Figure 1B,C and Figure 6A). Examination whether hit compounds from the synthesized derivatives are able to inhibit other highly pathogenic CoVs, such as SARS-CoV and MERS-CoV, as well as low pathogenic α- or β-CoV strains remains a strategic option for discovery of pan-coronavirus therapies.

On the basis of isobologram analysis, it was additionally verified that gemcitabine can achieve synergism when combined with alternative nucleoside analogue, remdesivir (Figure 7A,B). This finding means that the two nucleoside analogues tested participate in the antiviral machinery by targeting different proteins or by binding to different sites of a same target molecule in a non-competitive way. Similarly, it has been proposed that a combination of gemcitabine and ribavirin yields a synergistic effect in inhibition of coxsackievirus B3 in vitro [32]. Even though they are classified into the nucleoside analogues, their mode-of-antiviral actions can be non-identical with an assumption that remdesivir acts by binding the active site of viral nsp12 [15], whereas gemcitabine blocks host DNA synthesis mainly [33] and interferes with RNA synthesis by incorporation into cellular and/or viral RNA [25]. As a further study to prove this hypothesis, it is necessary to investigate whether gemcitabine directly recognizes the nsp12 subunit by three dimension structure analysis or enzymatic activity assay using purified, reconstituted replication-transcription complex. Most of all, considering the limitation of gemcitabine in a long term treatment as an antiviral agent, it could be a desirable way to reduce its treatment concentration through a synergistic combination with remdesivir or other promising drug candidates.

Western blot analysis and quantitative RT-PCR revealed that gemcitabine is active against SARS-CoV-2 infection in ACE2-expressing cell lines, either Vero cells or Calu-3 cells. However, in a comparative analysis, it was observed that gemcitabine is consistently effective in both cell lines, while remdesivir is more potent in Calu-3 cells rather than in Vero cells (Figure 3 and Figure 6). This cell line-dependent variation in antiviral activity of remdesivir seems to be primarily attributed to discrepancy in its pharmacokinetic properties. Pruijssers et al. previously reported that inhibition of SARS-CoV-2 by remdesivir is 2 to 6-fold more potent in Calu-3 cells than in Vero cells [34]. Being related to this, it is expected that reduced antiviral activity of remdesivir compared to gemcitabine in Vero cells might be associated with inefficient metabolism of the nucleoside prodrug, remdesivir, like sofosbuvir, in Vero cells [35]. Accordingly, additional synthesis of phosphoramidate prodrugs of gemcitabine or 2FdC or their derivatives might be a plausible approach for enhancement of cell membrane permeability and consequently improvement of antiviral efficacy in human lung cells [36]. In summary, this study provides information on antiviral activity of gemcitabine and 2FdC in vitro, that may be helpful for designing novel nucleoside-based antiviral compounds and ultimately for controlling the ongoing COVID-19 pandemic and preventing its reemergence.

## 4. Materials and Methods

### 4.1. Cells, Human Organoids, Viruses and Antiviral Compounds

Vero CCL-81 (Vero) cells and Calu-3 cells (American Type Culture Collection, Rockville, MD, USA) were maintained in Dulbecco’s modified Eagle’s medium (DMEM; Hyclone, South Logan, UT, USA) and in Eagle’s minimal essential medium (EMEM; Corning, Manassas, VA, USA), respectively, supplemented with 10% fetal bovine serum (FBS; Atlas Biological, Fort Collins, CO, USA) at 37 °C.

SARS-CoV-2 (BetaCoV/Korea/KCDC-03/2020) was provided by the Korea Centers for Disease Control and Prevention) and amplified in Vero cells through three passages in DMEM in the absence of serum, and aliquots were stored at −80 °C until use. Viral titer was determined by plaque assay [37]. All experiments with infectious SARS-CoV-2 were performed in a biosafety level 3 facility in KRICT.

Gemcitabine (≥98%) and 2FdC (97%) were purchased from Sigma-Aldrich (Saint Louis, MO, USA) and Combi-Blocks (San Diego, CA, USA), while remdesivir (99.7%) was synthesized by ST Pharm Co., Ltd. (Seoul, Korea), according to a previous report [38]. The identity and purity of remdesivir was analyzed by nuclear magnetic resonance spectroscopy (NMR) and mass spectrometry.

### 4.2. Cell Viability Measurement

Vero cells were seeded at a density of 2 × 10^4^ cells per well in 96-well plates, while Calu-3 cells were done at a density of 5 × 10^4^ cells per well. On the next day, cells were treated with increasing concentrations of gemcitabine for 24 and 48 h. Cell viability was measured by using MTT according to our previous report [39]. As an alternative way, cytotoxicity of gemcitabine to Vero cells using fluorescein diacetate [29]. After incubation of the cells with 100 μL of 30 μg/mL fluorescein diacetate solution for 30 min at 37 °C, fluorescence intensity reflecting cell viability was recorded at 485 (excitation) and 538 nm (emission).

### 4.3. Image-Based Antiviral Assay

Vero cells were seeded as mentioned above in the Section 4.2. After treatment of increasing concentrations of each compound (50 μL per well), they were infected with an equal volume of SARS-CoV-2 at a final multiplicity of infection (MOI) of 0.02 for 24 h. Cells were fixed and permeabilized with chilled acetone:methanol (1:3) solution at room temperature for 10 min. Viral S protein was probed using anti-S antibody (Cat. No., GTX632604; Genetex, Irvine, CA, USA) and Alexa Fluor 488-conjugated goat anti-mouse IgG antibody (Invitrogen, Carlsbad, CA, USA), while cellular nuclei were stained with 4′,6-diamidino-2-phenylindole (DAPI; Invitrogen). Fluorescence images were quantitatively analyzed using an Operetta High-content Screening System (Perkin Elmer, Waltham, MA, USA) and the built-in Harmony software. Fifty percentage effective concentration (EC_50_) was calculated by determining a chemical concentration required for reducing viral S-derived green fluorescence intensity by 50%. Fifty percentage cytotoxic concentration (CC_50_) was done by measuring a chemical concentration required for reducing DAPI-stained nuclear blue fluorescence intensity by 50%. Selectivity index (SI) was defined as the ratio of CC_50_ to EC_50_.

### 4.4. Western Blot Analysis

Vero cells seeded at a density of 5 × 10^5^ cells per well in 6-well plates were cultured overnight, while Calu-3 cells at the same density were done for 2 days. They were independently infected with SARS-CoV-2 at an MOI of 0.001 (Vero cells) or 0.002 (Calu-3 cells) for 1 h at 37 °C. Unabsorbed virus was removed by washing with PBS, and cells were treated with the compounds diluted in FBS-free fresh media. At 24 h post-infection, cell lysates were harvested using M-PER buffer (Thermo Scientific, Waltham, MA, USA). The immuno-transferred membrane was probed with primary anti-S antibody followed by secondary horseradish peroxidase (HRP)-conjugated anti-mouse rabbit IgG (Invitrogen). Cellular β-actin was used as a loading control.

### 4.5. RT-PCR

Vero or Calu-3 cells were infected with SARS-CoV-2 followed by treatment of various concentrations of each compound as mentioned in the Section 4.4. On the next day, culture supernatants were harvested for viral RNA purification using QIAamp viral RNA mini kit (Qiagen, Hilden, Germany). SARS-CoV-2 RNA load was quantified using a real-time RT-PCR kit with an N gene-specific primer set (PCLMD™ nCoV one step RT-PCR kit; PCL Inc., Seoul, Korea) and a CFX96 Touch real-time PCR instrument (Bio-Rad, Hercules, CA, USA).

### 4.6. Time-of-Addition

It was performed according to a previous report [40]. Briefly, Vero cells (1 × 10^5^ cells per well in 24-well plates) were successively but independently treated with 10 μM of gemcitabine at −3, 0, 3, 6 and 9 h from the SARS-CoV-2 infection (0.01 MOI) for 3 h, in which treatment with remdesivir at a same concentration was used as a control. At 20 h post-infection, culture supernatants were harvested to measure viral RNA titers.

### 4.7. Competition Assay with rNPTs

Vero cells seeded in 6-well plates were infected with SARS-CoV-2 at an MOI of 0.001 for 1 h. After washing out unabsorbed virus with PBS, they were incubated with 1, 10 and 100 μM of each rNTP (Invitrogen) in the absence or presence of 1 μM gemcitabine for 24 h. Cell culture supernatants and lysates were harvested for RT-PCR and western blot analysis.

### 4.8. Isobologram Analysis

Combination effect was tested according to previously established methods [39,41]. Given EC_50_ values of gemcitabine, 2FdC and remdesivir, either gemcitabine or 2FdC was mixed with remdesivir at various ratios of 5:0 (gemcitabine or 2FdC alone), 4:1, 3:2, 2:3, 1:4 and 0:5 (remdesivir alone), followed by preparation of 2-fold serial dilutions. Using SARS-CoV-2-infected Vero cells (MOI, 0.02), dose response curves were created to determine EC_50_ values of the mixture components at each combination ratio as well as their 50% fractional inhibitory concentrations (FIC_50_s). The sum of the FIC_50_s (ΣFIC_50_) of two compounds, e.g., compound A and compound B, was calculated according to an equation: ΣFIC_50_ = (EC_50_ of compound A in a combination/EC_50_ of compound A treated alone) + (EC_50_ of compound B in a combination/EC_50_ of compound B treated alone). When mean of ΣFIC_50_s was lower than 0.8, their interaction was classified as synergistic.

### 4.9. Statistical Analysis

Statistical analyses were performed by unpaired, two-way ANOVA *t*-test according to the Sidak’s multiple comparison method using GraphPad Prism version 8 (GraphPad Software Inc., La Jolla, CA, USA). *P* values lower than to 0.05 were considered statistically significant. * *p* < 0.05; ** *p* < 0.01; *** *p* < 0.001; **** *p* < 0.0001.

## Data Availability

Not applicable.
